# Effects of pH and Metal Ions on the Hydrothermal Treatment of Penicillin: Kinetic, Pathway, and Antibacterial Activity

**DOI:** 10.3390/ijerph191710701

**Published:** 2022-08-27

**Authors:** Qiaopan Zhang, Dongze Niu, Shensheng Ni, Wenying An, Chunyu Li, Taoli Huhe, Chongqing Wang, Xingmei Jiang, Jianjun Ren

**Affiliations:** 1 Changzhou Key Laboratory of Biomass Green, Safe & High Value Utilization Technology, Institute of Urban and Rural Mining, Changzhou University, Changzhou 213164, China; 2 School of Environmental and Safety Engineering, Changzhou University, Changzhou 213164, China; 3 Beijing General Station of Animal Husbandry, Beijing 100101, China; 4 Bijie Institute of Animal Husbandry and Veterinary Sciences, Bijie 551700, China

**Keywords:** penicillin, hydrothermal treatment, degradation products, antibacterial activity, kinetics

## Abstract

Antibiotic residues lead to the risk of resistance gene enrichment, which is the main reason why penicillin mycelial dreg (PMD) is defined as hazardous waste. Hydrothermal treatment (HT) is an effective method to treat penicillin mycelial dreg, but the degradation mechanism of penicillin is unclear. In the study, we researched the effects of pH (4–10) at 80–100 °C and metal ions (Mn^2+^, Fe^2+^, Cu^2+^, and Zn^2+^) at several concentrations on the HT of penicillin, identified the degradation products (DPs) under different conditions, and evaluated the antibacterial activity of hydrothermally treated samples. The results show that penicillin degradation kinetics highly consistent with pseudo-first-order model (R^2^ = 0.9447–0.9999). The degradation rates (*k*) at pH = 4, 7, and 10 were 0.1603, 0.0039, and 0.0485 min^−1^, indicating acidic conditions were more conducive to penicillin degradation. Among the four tested metal ions, Zn^2+^ had the most significant catalytic effect. Adding 5 mg·L^−1^ Zn^2+^ caused 100% degradation rate at pH = 7 after HT for 60 min. Six degradation products (DPs) with low mass-to-charge (*m*/*z* ≤ 335) were detected under acidic condition. However, only two and three DPs were observed in the samples catalyzed by Zn^2+^ and alkali, respectively, and penilloic acid (*m*/*z* = 309) was the main DPs under these conditions. Furthermore, no antibacterial activity to *Bacillus pumilus* was detected in the medium with up to 50% addition of the treated samples under acidic condition. Even though acid, alkali, and some metal ions can improve the degradation ability of penicillin, it was found that the most effective way for removing its anti-bacterial activity was under the acidic condition. Therefore, resistance residue indicates the amount of additive in the process of resource utilization, and avoids the enrichment of resistance genes.

## 1. Introduction

Antibiotic contamination has been widespread across the globe due to the overuse of antibiotics and inappropriate disposal of medical and pharmaceutical wastes [1,2]. Too much environmental release of antibiotics induces drug-resistant bacteria and then poses great threats to human health. Penicillin is one of the most widely used antibiotics, accounting for more than 80% of all antibiotics produced in China [3]. During its production process, lots of penicillin mycelial dreg (PMD), which is a bio-waste containing a large amount of organic substrate for fungal culture, equivalent to 30–50 times of penicillin itself, is produced [4]. Inappropriate disposal of PMD not only causes environmental pollution but also induces drug-resistance in bacteria due to the residual antibiotics [5]. Given the potential risks, China listed antibiotic mycelial dreg as hazardous waste in 2008, which limits the development of related pharmaceutical enterprises [6]. 

Reported methods for treating antibiotic mycelial dregs include pyrolysis, composting, and hydrothermal treatment (HT). Pyrolysis not only degrades residual antibiotics efficiently but also can convert the organic wastes into biochar and/or bioenergy [7,8,9], but the low economic efficiency and high carbon emission restrict its large-scale application. Although composting is an environment-friendly and economical method to treat organic wastes, residual antibiotics would potentially select for antibiotic resistance genes (ARGs) in bacteria [10]. To eliminate the potential risks in enriching ARGs and reduce the cost of the PMD treatment some researchers tried to degraded residual antibiotics in PMD by HT at subcritical temperatures, in which water generates hydronium and hydrated hydroxyl ions [11], and then used the treated PMD as raw material for composting [12] and methane production [13,14]. The results of previous studies proved that HT plays an important role in the disposal of PMD, but the main factors affecting degradation kinetics and safety of degradation products are still unclear.

Generally, the degradation of penicillin initiates from the opening of the four-membered lactam ring, which can be facilitated by some environmental factors [15]. It is reported that Zn^2+^ increased the degradation rate of penicillin caused by enzyme or photocatalysis in various aqueous solutions [16,17], and the adsorption of Zn^2+^ on the surface of goethite can also accelerate the hydrolysis of penicillin [18]. Fe^3+^ [19] and Mn^2+^ [20] promote the hydrolysis of penicillin, whereas Cu^2+^ can promote both the hydrolysis and oxidation process [21]. In addition, varying pH and temperature conditions also significantly affect the hydrolysis rate of penicillin antibiotics [22]. However, most of the studies focus primarily on the degradation in environmental waters under mild conditions; the effects of metal ions and pH on hydrothermal treatment penicillin and antibacterial activity of degradation products are currently unknown.

In this study, we determined the hydrothermal degradation kinetics of penicillin under different pH, temperature, and metal ions, identified the degradation products using liquid chromatography tandem mass spectrometer (LC-MS), and evaluated the antibacterial activity of the degradation products under different concentration.

## 2. Materials and Methods

### 2.1. Reagents and Bacterial Strain

Penicillin G potassium salt (C_16_H_18_KN_2_O_4_S, purity > 98%) was provided by Xinjiang Chuanning Biotechnology Co., Ltd. (Yining City, China). Buffer solution included phosphoric acid (H_3_PO_4_), disodium hydrogen phosphate (Na_2_HPO_4_·12H_2_O), and sodium dihydrogen phosphate (NaH_2_PO_4_·2H_2_O). The ion concentration in the reaction system was adjusted with copper nitrate (Cu(NO_3_)_2_·3H_2_O), zinc sulfate (ZnSO_4_·7H_2_O), manganese sulfate (MnSO_4_·H_2_O), and ferrous sulfate (FeSO_4_·7H_2_O). Acetonitrile, methanol, and formic acid (chromatographic grade) were used as mobile phase of liquid chromatogram. Unless declared, all the chemical reagents were analytical grade and purchased from Changzhou Runyou Petrochemical Co., Ltd. (Changzhou, China). 

*Bacillus pumilus* was also provided by Xinjiang Chuanning Biotechnology Co., Ltd., in a sealed cryopreservation tube and stored at −80 °C until use. Solid and liquid lysogeny broth (LB) media used to cultivate bacteria were same to previous reports [23,24]. Bacterial strain was passaged at least twice before use, for about 12 h each time. 

### 2.2. Hydrothermal Treatment

Experiments for different pH, temperature, and ionic concentration were conducted as a batch reaction. Firstly, 100 mL phosphate buffer solutions at pH 4, 6, 7, 8, and 10 in 250 mL Erlenmeyer flask were heated to required temperature (80, 90, and 100 °C) in a water bath. The initial penicillin concentration in the reaction system was adjusted to 100 mg·L^−1^ using stock solution (10 g·L^−1^). After 0, 5, 10, 15, 20, 30, 40, 60, 90, 120, 150, 180, and 240 min, 10 mL of the sample solution was neutralized with a small amount of highly concentrated NaOH (3 mol/L) or HCl (3 mol/L), and chilled in a 0 °C ice water to stop the reaction for further analysis. Using a similar method, penicillin was hydrothermally degraded with different ionic concentrations (0–10 mg·L^−1^) of Mn^2+^, Fe^2+^, Cu^2+^, and Zn^2+^ in a phosphate buffer solution at pH 7. All experiments were performed in triplicate.

### 2.3. Analytical Methods

Penicillin concentration was quantified using a Thermo Fisher series ultra-high Phase Liquid Chromatography (Ultimate3000, Thermo Fisher Scientific, Waltham, MA, USA) fitted with a reversed-phase C18 analytical column (1.8 μm, 250 mm × 4.6 mm; Yuexu Technology Ultimate XB-C18, Shanghai City, China) [24]. The mobile phase consisted of acetonitrile and formic acid (0.1%) in a 1:1 ratio with a flow rate of 1.0 mL·min^−1^ [25]. The column temperature, detection wavelength, injection volume, and total running time were 30 °C, 215 nm, 10 μL, and 7 min, respectively.

Penicillin degradation products were measured using a Waters series ultra-performance liquid chromatography tandem mass spectrometer (Xevo TQ-S, WATERS, Milford, MA, USA) equipped with electrospray ionization and a C18 analytical column (1.7 μm, 50mm × 2.1 mm; Waters ACQUITY^®^UPLCBEH C18) [14]. Methanol and formic acid (0.1%) were used as mobile phases [26]. The total flow rate and injection volume were 0.5 mL/min and 10 μL, respectively. The mass spectrometer was operated in positive ion full scan mode (100–400 *m*/*z*). The degradation products were deduced from the change in the peak area using MassLynx, and named according to their relative molecular mass and mass-to-charge ratio (*m*/*z*) using ChemDraw Ultra 20.0 software package (Cambridge, MA, USA).

### 2.4. Modelling of Penicillin Degradation

The rate constant (*k*) of the reaction was obtained according to pseudo-first-order model (Equation (1)).

(1) $$Ln\frac{C_{0}}{C}=kt$$

*C*_0_, initial penicillin concentration (mg·L^−1^); *C*, penicillin concentration at time *t* (mg·L^−1^); *k*, reaction rate constant (min^−1^); *t*, reaction time (min).

Activation energies of penicillin degradation under different pH were calculated according to Arrhenius equation (Equation (2)), which is an important thermodynamic parameter describing the degree to which the rate constant exponentially changes due to temperature.

(2) $$Lnk=-\frac{E_{a}}{RT}+Ln\left(A\right)$$

*E_a_*, activation energy (J·mol^−1^); *R*, molar gas constant (8.3141 J·K^−1^ mol^−1^); *T*, Kelvin temperature scale (K); *A*, pre-exponential factor.

A (Equation (3)) also known as the apparent frequency factor, which is determined only by the nature of the reaction.

(3) $$A=\frac{k_{B}T}{h}\times e^{\frac{\Delta S^{\neq }}{R}+1}$$

*k_B_*, Boltzmann constant (1.380649 × 10^−23^ J·K^−1^); *h*, Planck’s constant (6.62607015 × 10^−34^ J·s^−1^); Δ*S^≠^*, activation entropy (J·mol^−1^); *R*, molar gas constant (8.3141 J·K^−1^ mol^−1^).

Activation entropy is the kinetic process quantity. This value is similar to the Δ*S^≠^* calculated by the Eyring equation (Equation (4)) in the transition state theory.

(4) $$Ln\frac{k}{T}=\Delta H^{\neq }\times \left(-\frac{1}{RT}\right)+Ln\frac{k_{B}}{h}+\frac{\Delta S^{\neq }}{R}$$

Δ*H^≠^*, activation enthalpy (J·mol^−1^), which is the increment in standard enthalpy in the process of the reactant forming the activated complex. Δ*S^≠^*, activation entropy (J·mol^−1^), which is the increment of the standard entropy in the process of the reactant forming the activated complex.

*f* is the average hydrolysis rate factor (Equation (5)) for a 10 °C change in temperature using data collected at 80 °C and 100 °C.

(5) $$f=exp(\frac{Lnk_{100}-Ln(k_{80})}{2})$$

Half-life (*t*_1/2_) represents the time required for the compound to degrade to 50% of the initial concentration and is calculated according to Equation (6).

(6) $$t_{1/2}=\frac{Ln2}{k}$$

### 2.5. Antibacterial Activity of Penicillin and Its Degradation Products

Turbidimetric method was used to evaluate the antibacterial activity of the penicillin hydrothermal degradation products [27]. Penicillin and its degradation solutions were sterilized using filter membrane in an ultra-clean bench (Zhicheng Analytical Instrument Manufacturing Co., Ltd., Shanghai, China) to preclude any influence from other external microorganisms. The minimum inhibitory concentration (MIC) of penicillin was evaluated firstly at 0.5, 1.0, and 2.0 mg·L^−1^, and 1% (*v*/*v*) *B. pumilus* culture solution were inoculated in the LB medium. Similarly, 10%, 30%, 50%, and 70% penicillin degradation liquids (100 mg·L^−1^) after HT under acid, Zn^2+^, and alkali catalysis were added in 2- and 10-fold concentrations of LB medium to determine their antibacterial activity. A 48-well plate was used to detect the OD_600_ of each hole in a growth curve meter (FLUOstar Omega Micro-GCM, BMG LABTECH, Ortenberg, Germany) at 35 °C, 300 rpm.

## 3. Results and Discussion

### 3.1. Degradation Characteristics of Penicillin under Neutral Condition

**Figure 1:**
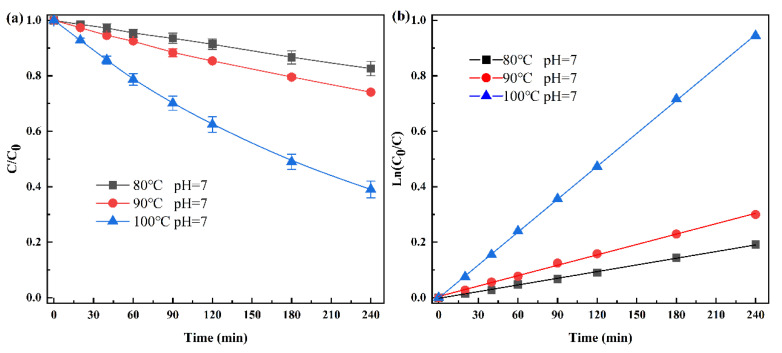
Degradation kinetics (**a**) and pseudo-first-order model (**b**) of penicillin at pH = 7 and different temperature during hydrothermal treatment. (*C*, concentration at time t; *C*_0_, initial concentration; Experiment condition, *C*_0_ = 100 mg·L^−1^).

Hydrothermal treatment under neutral or slightly acidic condition has been used to degrade residual antibiotics in PMD in industrial production [5,14,28], so we performed a preliminary experiment at pH 7 to determine the degradation kinetic of penicillin. As shown in Figure 1a, the degradation rate of penicillin is proportional to temperature, mainly attributing to that temperature is an important parameter for degrading target pollutants in HT. Therefore, increasing temperature concomitantly increases the number of activated molecules and strengthens the effective collision [4,29]. Although significant decrease in penicillin was observed in the process, the degradation rate of penicillin was only about 50% after 180 min HT at 100 °C, which is lower than previous report in PMD under identical conditions [5]. It is known that PMD has slightly lower pH and several metal ions, so the differences in degradation efficiency can be caused by some environmental factors. Furthermore, the degradation kinetics corresponds well with the pseudo-first-order model (Equation (1), R^2^ = 0.99, Figure 1b), which is similar to the hydrothermal degradation process of penicillin at 0–52 °C [30]. Above all, the hydrothermal degradation of penicillin in neutral buffer solution is slower than that in PMD and the process follows pseudo-first-order model.

### 3.2. Effects of pH on Penicillin Degradation at Different Temperatures

**Figure 2:**
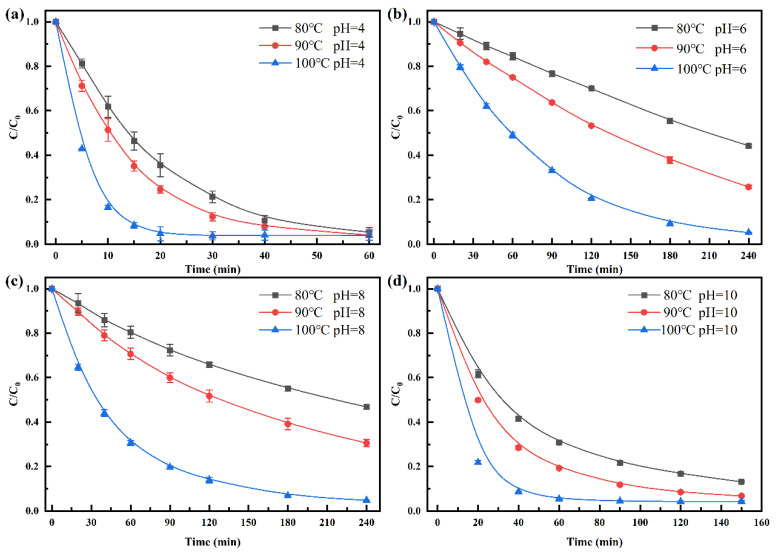
Effects of temperature and pH on the degradation kinetics of penicillin. ((**a**–**d**) treated under the condition of pH 4, 6, 8, and 10, respectively; *C*, concentration at time t; *C*_0_, initial concentration; Experiment condition, *C*_0_ = 100 mg·L^−1^).

The degradation kinetics of penicillin at different pH and temperatures are shown in Figure 2. Although penicillin antibiotics were alkali-labile [22], penicillin under acidic conditions were more favorable for the degradation of penicillin than alkaline condition during the HT, and the degradation rate increased with temperature raising. After 20 min of reaction, penicillin was completely degraded at pH 4 and 100 °C In comparison, the degradation rate of penicillin at pH 6, 8, and 10 were about 20%, 35%, and 80%, respectively. Time required for the complete degradation of penicillin at pH 6, 8, and 10 was 240, 180, and 60 min, respectively, which was 11 times, 8 times, and 2 times longer than that at pH 4. Wang et al. (2020) reported that residual antibiotics in PMD were completely degraded after HT at 100 °C for 2 h [5]. Generally, the pH of PMD was about 6, meaning penicillin is degraded at a higher rate in PMD than in phosphate buffers. The difference in degradation rate of penicillin at equal pH suggests the influence of other factors besides pH and temperature in the industrial HT process PMD, which potentially accelerate the degradation of penicillin.

Regression analysis revealed that the degradation kinetics of penicillin also fitted to pseudo-first-order model (R^2^ = 0.9447–0.9999, Table 1). Among the samples, the reaction rate constant *k* of penicillin degradation at same temperature was as follows: *k_pH_*_=4_ > *k_pH_*_=10_ > *k_pH_*_=6_ ≈ *k_pH_*_=8_ > *k_pH_*_=7_, indicating that penicillin was hydrolyzed much faster under acidic and alkaline conditions than under neutral conditions. Lu et al. (2008) also reported the higher degradation rate of penicillin under acidic conditions [31], where, at 5–50 °C, the reaction rate constant (*k*) of different pH was as follows: *k_pH_*_=4_ ≈ *k_pH_*_=10_ > *k_pH_*_=5_≈ *k_pH_*_=9_ > *k_pH_*_=6_ > *k_pH_*_=6.5_ ≈ *k_pH_*_=7.5_ > *k_pH_*_=7_. Conversely, the other β-lactams, such as ampicillin, cefthiophene, and cefoxitin, is more unstable under alkaline conditions than under acidic conditions [22]. This explains why penicillin is not suitable for direct oral administration [32].

Activation energy (*E_a_*), activation entropy (Δ*S^≠^*) and activation enthalpy (Δ*H^≠^*) represent the energy requirement for a chemical reaction [33]. Table 2 shows the *E_a_*, Δ*S^≠^*, and Δ*H^≠^* calculated using the Arrhenius equation and the Eyring equation. In particular, the parameters had highest value at pH 7, indicating that more energy was required for penicillin degradation under this condition. According to previous studies on penicillin [31] and other β-lactam compounds [22], such as ampicillin, cefotaxime, and cefoxitin, the activation energy is also maintained at about 80 kJ·mol^−1^, suggesting that all these compounds undergo almost the same reaction including the hydrolysis of β-lactam ring. The energy required at pH 4 and 10 is less than that at pH 7 by about 26–37 kJ·mol^−1^. At the same time, the effect of temperature on the rate of penicillin hydrolysis under different pH conditions is different. For example, temperature has the least effect on the reaction under pH 4 condition, because its *f* value is 1.77. Generally, *f* means the increased times of reaction rate when temperature was increased by every 10 °C, and the value of most chemical reactions is 2.5 [34]. In contrast, pH 7 had the highest effect, with *f* value as high as 4.87. Smaller activation energy under acid-catalyzed conditions further indicated the instability of penicillin under acidic conditions.

### 3.3. Effect of Metal Ion Species and Concentration on the Degradation Kinetics of Penicillin 

**Figure 3:**
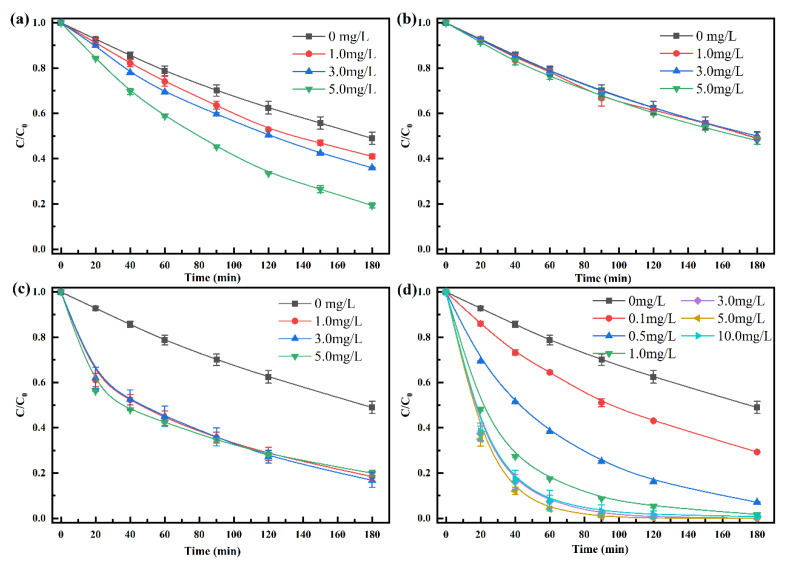
Effects of metal ions at different concentrations on the degradation kinetics of penicillin ((**a**–**d**), Mn^2+^, Fe^2+^, Cu^2+^, and Zn^2+^, respectively; *C*, concentration at time t; *C*_0_, initial concentration; Experimental condition, *C*_0_ = 100 mg·L^−1^, pH = 7, Temperature = 100 °C).

Figure 3 shows the degradation kinetics of penicillin with Mn^2+^, Fe^2+^, Cu^2+^, and Zn^2+^ at different concentrations. Increasing Mn^2+^ concentration increased the degradation rate of penicillin. For example, when the concentration was increased from 0 mg·L^−1^ to 5.0 mg·L^−1^, the degradation rate of penicillin increased by 30% after 180 min (Figure 3a). Generally, Fe^2+^ also can promote the hydrolyzation of β-lactam ring [19], but we did not find Fe^2+^ had positive effect on penicillin during HT process (Figure 3b). This can be attributed to the oxidation of Fe^2+^ into Fe^3+^ and Fe(OH)_3_ precipitation during the hydrothermal process and the complexation of Fe^3+^ with phosphate buffer solution [35]. Although the degradation rate of penicillin with Cu^2+^ is relatively fast in the first 20 min, the difference disappeared in the subsequent reaction (Figure 3c). Interestingly, we found that the reaction solution with Cu^2+^ first appeared green and then turned red as the reaction progressed. Generally, Cu^2+^ has both hydrolytic and oxidative effects in the natural environment [21,36], the changes in degradation rate and color indicates that most Cu^2+^ was converted to CuO during the heating process.

Among the four metal ions, Zn^2+^ had the highest catalytic efficiency in the hydrothermal degradation of penicillin. When Zn^2+^ concentration reached 5.0 mg·L^−1^, the degradation rate after 60 min was close to 100% (Figure 3d). At the same time, it should also be noted that the degradation rate of penicillin does not always increase with increasing Zn^2+^ concentration. When the concentration reached 3 mg·L^−1^, the degradation rate of penicillin tended to be stable. In addition, when Zn^2+^ concentrations were 3, 5, and 10 mg·L^−1^, the degradation rate was not significantly different (*p* > 0.05), which can be attribute to that the catalytic degradation is also dependent on the penicillin concentration. Above all, both Mn^2+^ and Zn^2+^ can promote penicillin degradation during HT process because both cations can degrade the β-lactam ring by complexing with the carboxyl group and tertiary nitrogen of β-lactam antibiotics [18,20], and Zn^2+^ had the most significant catalytic effect followed by Mn^2+^.

### 3.4. Degradation Products and Pathway of Penicillin under Different Conditions

**Figure 4:**
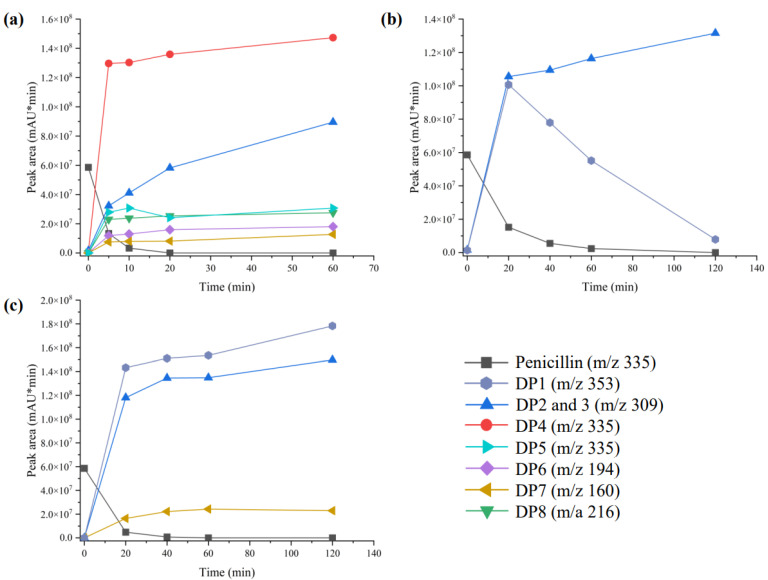
Peak area of penicillin and its degradation products during HT with acid ((**a**), pH = 4), Zn^2+^ ((**b**), C_Zn_ = 5 mg·L^−1^, pH = 7), and alkali ((**c**), pH = 10). (Experimental condition: *C*_0_ = 100 mg·L^−1^; Temperature = 100 °C).

**Figure 5:**
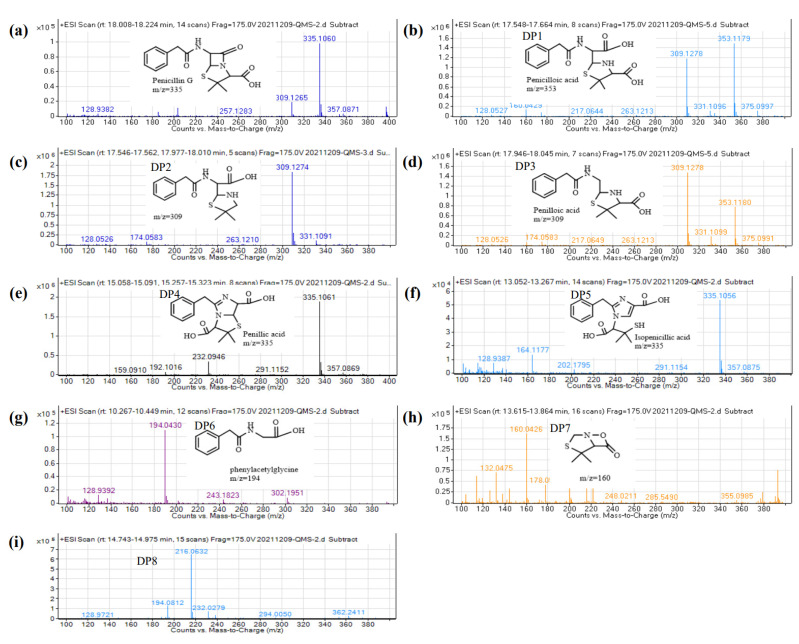
Mass spectrum of penicillin and its degradation products after hydrothermal treatment under different conditions ((**a**), The mass spectrum of penicillin G; (**b**–**i**), The mass spectrum of DP1, DP2, DP3, DP4, DP5, DP6, DP7, and DP8).

To determine the degradation mechanism of penicillin under different conditions, samples treated with acid (pH = 4), alkali (pH = 10), and Zn^2+^ (5 mg·L^−1^) were analyzed using LC-MS. Penicillin and eight degradation products were detected in hydrothermally treated samples, and one intermediate product was deduced (Figure 4). Three and two chemicals had equal mass-to-charge ratios (*m*/*z*), which were 335 and 309, respectively, but the diverse retention times indicate they are different compounds (Figure 5). The chemical (*m*/*z* 335) represents penicillin. DP1 (*m*/*z* 353), DP3 (*m*/*z* 309), DP4 (*m*/*z* 335), DP5 (*m*/*z* 335), and DP6 (*m*/*z* 194) were identified as penicilloic acid, penilloic acid, penillic acid, isopenicillic acid, and phenylacetylglycine, respectively. They also were detected under various agricultural conditions and sewage treatment by other researchers [17,26,37,38]. DP2 (*m*/*z* 309), DP7 (*m*/*z* 160), and DP9 (*m*/*z* 161) were named as 2-(5, 5-dimethylthiazolidin-2-yl)-2-(2-phenylacetamido) acetic acid, 4, 4-dimethyl-7-oxa-3-thia-1-azabicyclo [3.2.0] heptan-6-one, and 5,5-dimethylthiazolidine-4-carboxylic acid, respectively. They were reported in our previous study [14]. However, the structure of DP8 (*m*/*z* 216) is not yet known.

**Figure 6:**
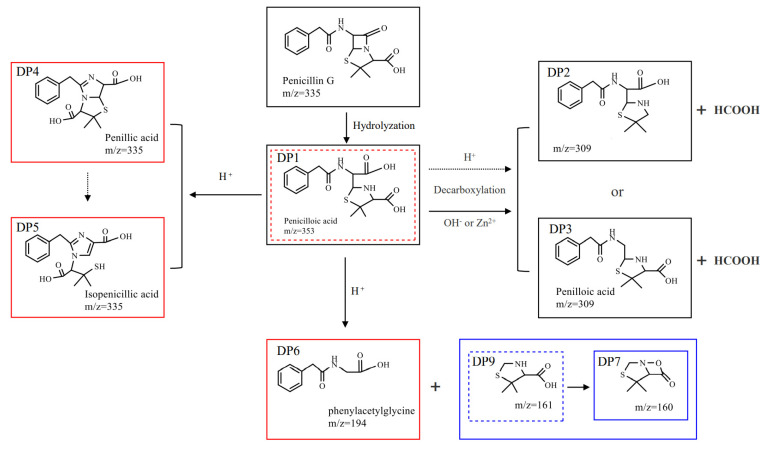
Proposed degradation pathway of penicillin during hydrothermal treatment with acid (pH = 4), Zn^2+^ (C_Zn_ = 5 mg·L^−1^, pH = 7), and alkali (pH = 10). (Experimental condition: *C*_0_ = 100 mg L^−1^; Temperature = 100 °C; dashed arrows, non-major pathways; dashed boxes, intermediates; red boxes, acid degradation products; blue boxes, acidic and alkaline degradation products; black boxes, common products).

Peak area changes of the degradation products are also shown in Figure 4. These products were more abundant under acidic conditions, up to six different kinds. Compared with samples containing Zn^2+^ and alkali, DP4 (*m*/*z* 335) and DP5 (*m*/*z* 335) were unique degradation product under acidic conditions, and DP4 was the main degradation product. The peak area of DP5 was about 85% less than that of DP4. Some researchers reported that a part of Penillic acid is converted to Isopenillic acid in anaerobic environments [37], so DP5 might be produced by DP4 (Figure 6). In addition, DP1 (*m*/*z* 353), DP6 (*m*/*z* 194), and DP8 (*m*/*z* 216) were also unique products under acidic conditions, and phenylacetylycine (DP6, *m*/*z* 194) is probably formed when 5,5-dimethylthiazolidine-4-carboxylic acid (DP9, *m*/*z* 161) is removed from penicilloic acid (DP1, *m*/*z* 353). Conversely, the peak aera of DP2 and DP3 (*m*/*z* 309) under acidic condition was lower than that in the groups with Zn^2+^ and alkali, and penicilloic acid was not directly detected in the former sample. It is reported that acidic conditions favor decarboxylation [39], we deduced that DP2 and DP3 were mainly formed by the decarboxylation of penicilloic acid. Although DP1, DP2, and DP3 were detected in both samples containing Zn^2+^ and alkali, the peak area of DP1 decreased sharply after 20 min in the former, indicating Zn^2+^ also contributes to the decarboxylation of penillic acid [17]. The evidence revealed that the decarboxylation reaction increased with the decrease in pH and penicillin has more degradation pathway and degrades more thoroughly under acidic condition.

### 3.5. Antibacterial Activity of Penicillin and Hydrothermally Treated Samples

**Figure 7:**
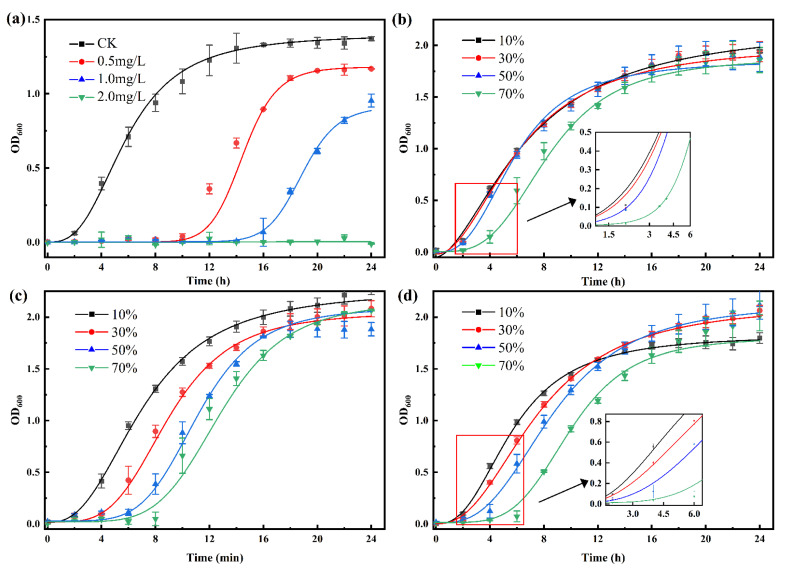
Antibacterial activities of penicillin (**a**) and its degradation solution at different concertation after hydrothermal treatment with acid ((**b**), pH = 4), Zn^2+^ ((**c**), pH = 7, C_Zn_ = 5 mg·L^−1^), and alkali ((**d**), pH = 10) under 100 °C.

Figure 7 presents the growth of *B. pumilus* in LB medium with different concentrations of penicillin and its hydrothermally treated solutions. As shown in Figure 7a, *B. pumilus* is very sensitive to penicillin. When 0.5 mg·L^−1^ penicillin was added in the medium, the time of entering the logarithmic growth phase was delayed by 8 h. The inhibition of penicillin to *B. subtilis* was also reported by other researchers [17], attributing to penicillin interferes with the cell wall synthesis. Figure 7b–d present the delay period increased with the proportion of the degradation solution, especially for the groups treated with Zn^2+^ at 70% addition, the logarithmic growth period is delayed by 6 h. Some researchers reported Zn^2+^ can promoted the inhibitory effect of penicillin on *B. pumilus* [40,41]. Based on the analysis of LC-MS, we deduced the delay period can be caused by some degradation products or residual penicillin. DP7 produced at pH = 4 has a lactam structure and may have some bacteriostatic effects, resulting in 2 h delay in reaching the logarithmic growth phase at 70% addition. DP1 is one of the main products at pH = 7 and pH = 10, which may also be the reason for the delayed logarithmic growth phase. However, in the cultivation with hydrothermally treated penicillin, *B. pumilus* can grow well and reach a stable stage within 24 h even when the addition ratio of the reaction solution reaches 70%, and no inhibitory action was observed in 10% addition of all the samples. Therefore, the use of PMD after hydrothermal treatment for subsequent utilization, such as composting, is very environmentally friendly.

## 4. Conclusions

The hydrothermal degradation of penicillin conforms to pseudo-first-order model. The degradation rate constant *k* of penicillin under different pH was as follows: *k_pH_*_=4_ > *k_pH_*_=10_ > *k_pH_*_=6_ ≈ *k_pH_*_=8_ > *k_pH_*_=7_, and it increased with the rising temperature. Among the tested metal ions, Zn^2+^ has the most significant catalytic effect, followed by Cu^2+^, Mn^2+^, and Fe^2+^. Samples treated under acidic condition have more degradation products and lower antibacterial activity than others. Further study should pay more attention to the degradation products of penicillin in PMD and their safety and fates during subsequent utilization.

## Figures and Tables

**Table 1:** Pseudo-first-order model parameter of penicillin degradation at different pH and temperature.

pH	Temperature (°C)	*k* (min^−1^)	R^2^	Approximated *t*_1/2_ (min)	Observed *t*_1/2_ (min)
pH = 4.	80	0.0514	0.9918	13.48	13.61
	90	0.0654	0.9942	10.59	10.14
	100	0.1603	0.9955	4.32	4.26
pH = 6	80	0.0032	0.9946	216.56	209.01
	90	0.0056	0.9975	123.75	131.92
	100	0.0127	0.9963	54.57	57.85
pH = 7	80	0.0008	0.9984	866.25	>240.00
	90	0.0012	0.9983	577.50	>240.00
	100	0.0039	0.9999	177.69	174.88
pH = 8	80	0.0032	0.9957	216.56	215.41
	90	0.0050	0.9952	138.60	127.74
	100	0.0126	0.9647	55.00	34.01
pH = 10	80	0.0169	0.9752	41.01	31.11
	90	0.0234	0.9713	29.62	20.21
	100	0.0485	0.9447	14.29	12.62

*k*, reaction rate constant; *t*_1/2_, half-life; R^2^, goodness of fit.

**Table 2:** Arrhenius coefficient at different pH calculated according to Arrhenius equation.

Items	pH				
	4	6	7	8	10
*E_a_* (kJ·mol^−1^)	60.80	75.36	86.37	74.82	64.50
*LnA* (min^−1^)	17.67	19.88	22.15	19.65	17.71
*f*	1.766	3.97	4.88	3.94	2.87
Δ*H^≠^*(kJ·mol^−1^)	57.78	72.35	83.35	71.80	61.48
Δ*S^≠^*(kJ·mol^−1^)	−107.98	−89.63	−70.69	−91.46	−107.62

*E_a_*, activation energy; *LnA*, pre-exponential factor; *f*, average hydrolysis rate factor for a 10 °C; Δ*H^≠^*, activation enthalpy; Δ*S^≠^*, activation entropy.

## Data Availability

Not applicable.
